# CSE regulates LINC000665/XBP-1 in the progress of pulmonary fibrosis

**DOI:** 10.18332/tid/175004

**Published:** 2023-12-18

**Authors:** Min Song, Qinxue Shen, Xiaoli Ouyang, Zijing Zhou, Hong Luo, Hong Peng

**Affiliations:** 1 Department of Pulmonary and Critical Care Medicine, The Second Xiangya Hospital, Central-South University, Changsha, China; 2 Research Unit of Respiratory Disease, Central-South University, Changsha, China; 3 Clinical Medical Research Center for Pulmonary and Critical Care Medicine in Hunan Province, Changsha, China; 4 Diagnosis and Treatment Center of Respiratory Disease, Central South University, Changsha, China

**Keywords:** pulmonary fibrosis, smoking, lncRNA LINC00665, XBP-1, cigarette smoke exposure

## Abstract

**INTRODUCTION:**

Cigarette smoking may impact the progression of idiopathic pulmonary fibrosis (IPF), and the intensity of smoking presents a dose-response association with IPF.

**METHODS:**

We retrospectively analyzed IPF patients diagnosed in our hospital from 2014 to 2018 and performed follow-up to confirm survival status and duration, and determine the effect of smoking on the prognosis of IPF. We retrieved information on IPF from a bioinformatics database to identify the differential expression of lncRNAs and proteins in smokers. Therefore, we explored and verified the mechanism by which cigarette smoke exposure (CSE) regulates LINC00665/XBP-1 involvement in pulmonary fibrosis through cell experiments. We clarified the mechanism between LINC00665 and XBP-1 through cellular and molecular experiments, and verified the inhibitory effect of silencing LINC00665 on pulmonary fibrosis by using a bleomycin (BLM)-induced pulmonary fibrosis model.

**RESULTS:**

We found that smokers with IPF had a poor prognosis compared with non-smokers. Both the expression of LINC00665 and XBP-1 in IPF lung tissue and smoker lung tissue were significantly upregulated, moreover, LINC00665 was higher in smoker IPF lung tissue than in smoker healthy people. Exposure to CSE could upregulate LINC00665/XBP-1 in lung fibroblast-to-myofibroblast transition. Cellular and molecular experiments showed that LINC00665 regulates the expression of XBP-1 by targeting miR-214-3p. LINC00665 expression, was significantly upregulated in BLM-induced mouse lung fibrosis tissues, and LINC00665 knockdown inhibited fibrogenesis in BLM-induced lung fibrosis.

**CONCLUSIONS:**

Our study found that the high expression of LINC00665 is involved in the pathogenesis of smoker IPF and that CSE may positively regulate LINC00665/XBP-1 to participate in lung fibroblast-to-myofibroblast transition. These findings help elucidate the pathogenesis of smoker IPF and may contribute to the development of new targeted drugs for IPF therapy.

## INTRODUCTION

Idiopathic pulmonary fibrosis (IPF) often has a progressive aggravation and poor prognosis with an unknown etiology, and there is no effective treatment thus far. Smoking-related exposure was found to be associated with the risk of IPF^1,2^, and in ever smokers, a relationship was observed between smoking pack-years and the risk of IPF^2^. We retrospectively analyzed the relationship between smoking exposure status and the survival time of IPF patients diagnosed in our hospital from 2014 to 2018 and found that smoker IPF patients had a poor prognosis compared with non-smokers. These findings inspired us to study the pathogenesis of smoking and IPF.

Long noncoding RNAs (lncRNAs) are RNAs that are transcribed from the genome but do not encode proteins and are important regulatory factors of various diseases. The function and mechanism of lncRNAs in IPF are also the focus of current research^3-5^. Numerous studies have proven that aberrant expression of lncRNAs is closely linked with the pathophysiology of IPF-related pulmonary fibrosis and plays an important role in the pathogenesis of IPF ^6,7^. Endoplasmic reticulum (ER) stress is involved in the development and progression of pulmonary fibrosis, and the ER stress-induced unfolded protein response (UPR^ER^) has been linked to pulmonary fibrosis^8^. UPR^ER^ dysregulation is associated with IPFrelated pulmonary fibrosis, so effective and specific compounds targeting the UPR pathway are considered potential therapeutic methods for fibrotic diseases^9,10^.

Accordingly, we searched the RNA sequencing database of IPF patients (GSE134692), and the results of data analysis revealed that the expression of LINC00665 and XBP-1 in lung tissues of IPF patients was substantially increased compared with that in normal tissues. We further found that the expression levels of LINC000665 and XBP-1 in smoker lung tissues were significantly higher than those in non-smokers, and LINC00665 was also significantly higher in smoker IPF lung tissues than in smoker healthy controls. The lung fibroblast-to-myofibroblast transition is a key link event in the pathogenesis of IPF-related pulmonary fibrosis. Previous studies have proven that LINC00665 can promote the malignant transformation of precancerous lesions and the migration and invasion of a variety of tumours^11-13^. However, the mechanism of LINC00665 in smoking-related pulmonary fibrosis has rarely been studied. In this study, we aim to ascertain the relationship between smoking, pulmonary fibrosis, LINC00665, and XBP-1. However, the regulatory role of cigarette smoke exposure (CSE) on LINC00665/XBP-1 in the process of pulmonary fibrosis has not been officially reported. In our study, we explored the mechanism of CSE on LINC00665 and XBP-1 in lung fibroblast-to-myofibroblast transition, confirmed the interaction between LINC00665 and XBP-1, and observed the inhibitory effect of silencing LINC00665 on pulmonary fibrosis in mice. Our overall objective is to investigate the mechanism of the effect of smoking on pulmonary fibrosis and to explore a new potential strategy for IPF.

## METHODS

### Study design and patients

This retrospective observational cohort study was conducted with IPF patients hospitalized in the Second Xiangya Hospital of Central South University) in Hunan, China, from January 2014 to October 2018. Institutional approval was provided by the Second Xiangya Hospital of Central South University Biomedical Research Ethics Committee (Hunan, China). Written informed consent was waived because of the retrospective observational design. All patient data were anonymously recorded to ensure confidentiality. Patients were admitted to the hospital with a diagnosis of IPF based on the 2011 IPF definition^14^. The diagnosis of IPF requires the following: 1) Exclusion of other known causes of interstitial lung disease (ILD); 2) The presence of a UIP pattern on high-resolution computed tomography (HRCT) in patients not subjected to surgical lung biopsy; and 3) Specific combinations of HRCT and surgical lung biopsy pattern in patients subjected to surgical lung biopsy.

Smoking-related information was collected from all patients and a telephone follow-up to inquire about their current living conditions was conducted. Never smokers were defined as those who smoked <100 cigarettes in their lifetime. The primary outcome was mortality.

### Statistical analysis of the Bioscience database

RNA sequencing of idiopathic pulmonary fibrosis lung and healthy control normal tissues was downloaded from GEO (www.ncbi.nlm.nih.gov/geo/query/acc.cgi?acc=GSE134692). The batch effect of data was removed by the R package *limma* and expression was log2CPM normalized. We visualized the distribution of data between the healthy control group and IPF patient group using the t-SNE method to perform quality control, which included 46 IPF and 26 normal lung tissues. The statistics of the two-group comparisons were compared with the Wilcoxon test, and the correlation method was Spearman’s test.

### Experimental pulmonary fibrosis model

*Animal preparation*

C57BL/6 mice (male, 6–8 weeks old) were acquired from the Experimental Animal Center of the Second Xiangya Hospital of Central South University. The mice were used for experiments and controls were fed for 1 week under the experimental conditions. Establishment of a bleomycin (BLM)-induced pulmonary fibrosis model in mice involved a lung fibrosis model established after intratracheal injection of BLM (5 mg/kg, Sigma) under anesthesia with intraperitoneal injection of 2% pentobarbital by volume weight. Mice were injected with normal saline as a control. According to the different treatment groups after 72 h, the transplantation group mice were intratracheally injected with 50 μL shLINC00665 (GenePharma, Shanghai), and the transplantation control group mice were injected with 50 μL shNC (GenePharma, Shanghai). The samples were collected 7, 14 and 28 days later.

### Extraction, isolation, identification, culture, and treatment of lung fibroblasts (LFBs)

C57BL/6 mice (male, 6–8 weeks old) were sacrificed under anesthesia and painless conditions, and their lungs were rapidly collected in a sterile state. After rinsing with PBS 3 times, the samples were cut into 1 mm^3^ tissue blocks, which were evenly spread at the bottom of a 10 cm petri dish and digested with 0.25% trypsin. The isolated cells were cultured in 10% bovine serum DMEM with 100 IU/mL penicillin and 100 IU/mL streptomycin in a humidified incubator at 37^o^C with 5% CO_2_ and a constant temperature for 2 to 3 days. When the cells reached 90% confluence, they were digested and subcultured. After 4 generations of culture, the cells showed typical spindle shapes. Vimentin was strongly positive in the cytoplasm, as demonstrated by immunocytochemistry, and the cells were confirmed to be fibroblasts for further experiments. LFBs were cultured at 37°C with 5% CO_2_. Recombinant TGF-β1 (10 ng/mL, Sigma) was used to establish a pulmonary fibrosis model *in vitro*. Cells were treated with an inducer of ER stress, thapsigargin (TG, 0.2 μM, T9033, Sigma) to regulate the expression of XBP-1 in lung fibroblasts^15^.

### Preparation of CSE

CSE was prepared as previously described^16-18^. One cigarette (Baisha, China Tobacco Hunan Industrial Corporation, Changsha, Hunan, China) burned yielded 25 mg of tar, 10 mg of CO and 0.1 mg of nicotine under a standard smoking regimen, and the smoke was passed through 20 mL of phosphate-buffered saline via a vacuum pump. This 100% CSE solution was adjusted to 7.2–7.4 and filtered through a 0.22 m membrane filter to remove large particles and bacteria before use. Then, CSE was diluted with PBS to obtain concentrations of 1% (0.1 M) and 10% (1.0 M) and these samples were used for the cell experiments within a period no longer than 30 min after preparation.

### Cell transfection

According to the lncRNA database, the gene sequence of LINC00665 was obtained, and double-stranded DNA and iRNA vectors were synthesized. Then, the double-digested lentivirus vector was linked to the cloned double-stranded DNA. The constructed vector and lentivirus packaging plasmid vector pcDNA3.1 were coinfected in 293T packing cells by Lipofectamine 2000, and the virus genome was generated. It was determined by the pore-release method. The prepared lentivirus vector was stored at -80°C. Plasmid construction and lentivirus packaging were completed by Hunan Pratze Biotechnology Co., Ltd.

### Immunofluorescence

The cells in each group were inoculated in 24-well plates at 1×10^4^ cells/well and cultured to 50% confluence. The original medium was discarded and replaced with serum-free basic medium for 24 h. We extracted the original culture medium, washed the cells with PBS for 3 times, fixed with 4% paraformaldehyde for 15 minutes, and washed with PBS for 3 times, 5 minutes each time. We added Triton X-100 (0.3%) to the membrane for 15 min, and washed with PBS for 3 times, 5 min each time. Goat serum was added for 1 h. The sealing solution was aspirated without rinsing, and mouse anti-rat α-SMA was added and incubated at 4°C overnight. The next day, the primary antibody was recovered, and the samples were rinsed with PBS 3 times for 5 min each. The immunofluorescent FITC-labelled goat anti-mouse IgG secondary antibody was added dropwise and incubated at room temperature for 1–2 h. The fluorescent secondary antibody was recovered, and then rinsed 3 times with PBS for 5 min each time. After rinsing with PBS, DAPI staining solution was added for 10 min at room temperature and then washed 3 times with PBS for 5 min each time.

### Relative luciferase activity

MiR-214-3p was predicted to be a potential biological target of LINC00665 by StarBase. The double luciferase reporter assay further confirmed its targeting relationship. The 3’ untranslated regions (UTR) of LINC00665 and miR-214-3p were identified, and then, plasmids containing potential miR-214-3p binding sites and LINC00665 wild-type (WT) or mutant (MUT) sites were cotransfected with miR-NC or miR-214-3p mimic. The LINC0065 WT group and LINC00665 MUT group were compared to verify whether miR-214-3p was the direct target of LINC00665 through relative luciferase activity. Based on the binding sequence of the target gene 3’UTR to miR-214-3p, the design was generated using the miRNABase database. The XBP-1 target gene fragment was synthesized by PCR, and the synthesized target gene fragment was cloned into the XhoI and NotI sites, while one end of the synthesized gene fragment was cloned into the pluC-UTR luciferase reporter gene expression vector. The plasmid was named pluC-XBP-1-3’UTR, and the construct was confirmed by sequencing. However, the seed region sequence was mutated by point mutation. The two groups of luciferase expression vectors were named. The luciferase reporter gene expression vectors were wild-type (XBP-1-WT) and mutant (XBP-1-MUT).

A dual luciferase assay kit (Promega) was used. Enzyme activity was determined according to the instructions. XBP-1 was cloned into a 3’UTR plasmid or a 3’UTR vector. Then, 293T cells were cotransfected with miR-214-3p mimics and controls. Twenty-four hours after transfection, the cells were collected in cold PBS solution, and 100 μL of cell lysate buffer was added to the cells. After centrifugation at 4°C, 20 μL of supernatant was added to 96-well plates, and 30 μL of LAR II was added to mix. Determination of firefly luciferase activity was performed on a microplate reader (F value). The luciferase activity (R value) of Renilla was determined by adding 30 μL of Stop & Glo reaction solution. The ratio of the F value to the R value reflected the comparative expression level of the reporter gene.

### RNA binding protein immunoprecipitation (RIP) assay

The binding relationship between endogenous LINC00665 and miR-214-3p was studied by the RIP method. The lysate transfected with LFB was treated with RIP buffer containing magnetic beads coupled with anti Ago2 antibody (Millipore, Billerica, MA, USA), and the negative IgG was used as a control. We washed the pellets with washing buffer, and incubate with 0.1% SDS/protease K to remove protein. Finally, real time quantitative PCR (qRT-PCR) was used to detect their expression levels.

### qRT‑PCR

Total RNA was extracted from lung tissue or cultured LFBs with TRIzol reagent (Invitrogen, USA). A reverse transcriptase kit was used to synthesize cDNA. An ABI 7500 Fast Real-Time PCR instrument (California, USA) was used to determine gene expression with SYBR Green I.

### Western blot analysis

After each group LFBs were treated at a certain concentration for 48 h, followed by a mixture of the BRIPA lysis buffer and a protease inhibitor cocktail (GenDEPOT; P3100, P3200) to prepare the whole cell proteins.

We measured the cell protein concentrations with a bicinchoninic acid assay kit. Protein samples were separated by sodium dodecyl sulfate-polyacrylamide gel electrophoresis and transferred to nitrocellulose membranes. We incubated the membrane with the following antigen recognition antibody at 4^o^C overnight: α-SMA, fibronectin, collagen I, E-cadherin and XBP-1,blocking with 5% nonfat milk later. Incubation of membrane with polyclonal anti rabbit/mouse or goat HRP binding secondary antibody (1:5000 dilution; LI-COR Biosciences, Lincoln, NE, USA) for 1 h at room temperature was done, after washing with Tris-buffered saline Tween. Next, we used Tris-buffered saline-Tween to wash the membranes 3 times, and using an ECL detection solution to detect. We used the Odyssey infrared imaging system (Gene Company, Beijing, China) to scan and quantify the band intensities.

### Statistical analysis

We used SPSS 22.0 (SPSS, Inc., Chicago,IL, USA) and GraphPad Prism 8.0 (GraphPad Software Inc. USA) to perform the data analysis. We used Kaplan-Meier plots and log-rank tests to compare the survival rate of each group. We calculated an independent predictor of mortality with stepwise binary logistic regression variables for the regression values of p<0.05 (one variable was entered when p<0.05, and one was deleted when p>0.10). The odds ratio (OR), p-value and 95% CI were used to represent the results. The data are expressed as the mean ± SEM, except where otherwise indicated. The Kruskal-Wallis test was used to determine the difference detection for different groups, and the Mann-Whitney U test was used to analyze the differences between individual variables from the 2 groups. A p<0.05 was considered significant.

## RESULTS

### The relationship between smoking status and survival rate in IPF patients, and statistical analysis of the Bioscience database

**Figure 1:**
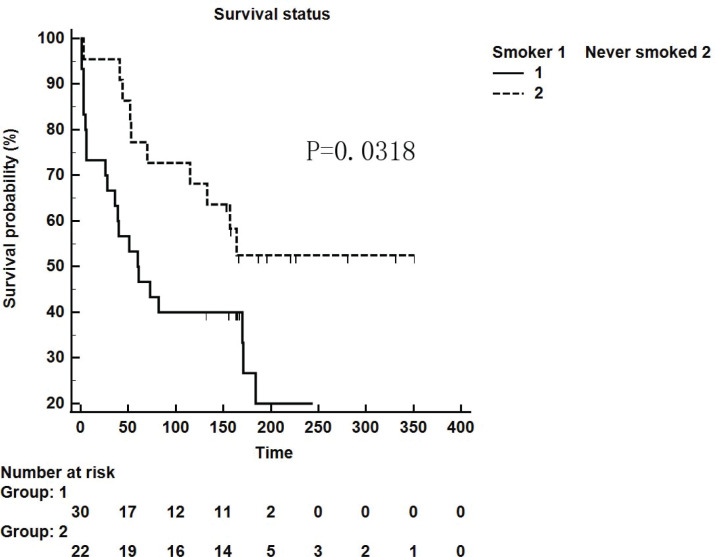
Kaplan-Meier survival curve for patients with IPF using the cut-off values for the smokers and never-smokers obtained by ROC analysis (log-rank test)

We retrospectively analyzed IPF patients hospitalized in the Department of Respiratory and Critical Care Medicine of Xiangya Second Hospital of Central South University from January 2014 to October 2018. There were 87 patients in total, 41 (47.13%) of whom had a smoking history. Furthermore, these patients were followed up and 25 patients were lost. Among 52 patients who were followed up successfully, the influence of smoking as a single factor on survival time was compared. Thirty smoker patients and 22 never smoker patients were followed up for survival time in weeks. It was found that the survival time of patients with a smoking history was shorter than that of non-smokers (p=0.0318, Figure 1).

The COX multivariable survival analysis beyond smoking with more complete data is shown in Table 1).

**Table 1:** Multivariable survival COX analysis of IPF patients beyond smoking

*Variables*	*Univariate analysis*		*Multivariate analysis*	
	*OR (95% CI)*	*p*	*OR (95% CI)*	*p*
Gender	1.37 (0.66–2.87)	0.3994		
Age	1.05 (0.78–1.91)	0.1471		
BMI (kg/m^2^)	1.02 (0.84–1.30)	0.1389		
PaO2 (mmHg, no oxygen at tranquilization)	0.95 (0.86–1.14)	0.0021	1.29 (1.10–1.51)	0.0043
SaO2 (%, no oxygen at tranquilization)	1.03 (0.80–1.81)	0.0635		
Acute exacerbation	3.36 (1.04–6.88)	0.0009	3.17 (1.64–6.81)	0.0134
No antifibrotic drugs used	2.18 (1.07–4.44)	0.0318	2.81 (2.02–4.31)	0.0141

BMI: body mass index. PaO2: arterial partial oxygen pressure. SaO2: pulse oxygen saturation.

The database includes 46 IPF and 26 normal lung tissues (Supplementary file Figure 1A). The Wilcoxon test was used for comparisons between the two groups, and Spearman’s test was used for correlations. The results of the data analysis showed that compared with that in normal lung tissue, the expression of LINC00665 in IPF lung tissues was obviously higher than that in normal healthy control lung tissues (p=0.000006*,* p<0.001) (Supplementary file Figure 1B), and the expression of XBP-1 in IPF lung tissue was significantly increased (Supplementary file Figure 1C, p=0.014). The expression levels of LINC00665 and XBP-1 in the former smoker lung tissues were significantly higher than those in the never smoker lung tissues (Supplementary file Figure 1D, p=0.018; and 1E, p=0.029). The expression level of LINC00665 in the former smokers’ IPF lung tissues was higher than that of the former smokers’ healthy controls (Supplementary file Figure 1F, p=0.037).

### CSE regulates the LINC00665/XBP‑1 in the transformation of pulmonary fibroblasts

The fibroblasts were cultured in the presence or absence of an increasing concentration of CSE (0.1 M, 1.0 M) for 48 h; immunocytochemical analysis of α-SMA protein expression showed that the fibroblasts treated with 1.0 M CSE for 48 h resulted in more obvious expression of α-SMA than that in the 0.1 M group. (Supplementary file Figure 2A). Then, we determined LINC00665 expression in the control group, 0.1 M CSE and 1.0 M CSE group at 24 h and 48 h time points respectively, and the expression of LINC00665 increased significantly in the 1.0 M CSE group compared to the 0.1 M CSE group at different time points (Supplementary file Figure 2B, p<0.05). We used shRNA targeting LINC00665 to knockdown the expression of LINC00665, and the effect of shRNA1 was more obvious (Supplementary file Figure 2C, p<0.05) thus, this shRNA was selected for use in follow-up experiments. We used western blotting to monitor α-SMA and XBP-1 protein expression levels in different groups and examined the effect of different combinations of CSE, shLINC00665 and shNC. The results revealed that the expression of α-SMA and XBP-1 was obviously upregulated after treatment with CSE alone and CSE combined with shNC group, but was obviously downregulated after transfection with shLINC00665 after CSE compared to treatment with CSE alone. (Supplementary file Figure 2D, p<0.05). The expression level of XBP-1 in LFBs was significantly increased with TG treatment alone. However, the expression level of α-SMA in fibroblasts was substantially decreased after treatment with shLINC00665, but could be reverted upwards after restoring XBP-1 levels by TG with shLINC00665 *in vitro* (Supplementary file Figure 2E, p<0.01; and 2F, p<0.05).

### LINC00665 regulated the expression of XBP‑1 by targeting miR‑214‑3p

We predicted that miR-214-3p was a potential biological target of LINC00665 by StarBase (http://starbase.sysu.edu.cn) and further confirmed its targeting relationship by a double luciferase reporter assay (Supplementary file Figure 3A). The 3’UTR loci of LINC00665 and miR-214-3p were identified, and then plasmids containing potential miR-214-3p binding sites and LINC00665 WT or MUT sequences were cotransfected with miR-NC or miR-214-3p mimic. The relative luciferase activity of the LINC0065 WT group and the LINC00665 MUT group was compared to verify whether miR-214-3p was the direct target of LINC00665 (Supplementary file Figures 3B and 3C). After TGF-β1 treatment of LFBs, the expression of miR-214-3p decreased compared with that in the control group. However, the expression of miR-214-3p in LFBs increased significantly after treatment with shLINC00665 (Supplementary file Figure 3D, p<0.01). Based on the binding sequence of the target gene 3’-UTR to miR-214-3p, the design was made using the miRNA Base database XBP-1 target sequence. The XBP-1 target gene fragment was synthesized by PCR. The results of double luciferase reporter gene analysis showed that miR-214-3p may bind to XBP-1 through the predicted binding site (Supplementary file Figures 3E and 3F). The expression of miR-214-3p in LFBs infected with miR-214-3p mimics was evidently higher than that in the control group (Supplementary file Figure 3G, p<0.01). These results indicated that miR-214-3p mimics can clearly improve the expression level of miR-214-3p, and the expression of miR-214-3p in LFBs transfected with miR-214-3p inhibitor was obviously lower than that in the inhibitor-transfected control group (Supplementary file Figure 3G, p<0.01). Moreover, the mRNA expression of XBP-1 in LFBs infected with miR-214-3p mimics was significantly lower than that in LFBs infected with mimics-NC, and the miR-214-3p inhibitor significantly increased the expression level of XBP-1 (Supplementary file Figure 3H, p<0.01). We used western blotting to monitor XBP-1 protein expression levels in LFBs with miR-214-3p mimics and inhibitors. The results revealed that miR-214-3p mimics obviously reduced the protein expression level of XBP-1 in cells compared with the control group, but miR-214-3p inhibitors obviously increased the protein expression level of XBP-1 (Supplementary file Figure 3I, p<0.01). We used western blotting to monitor XBP-1 protein expression levels in different groups and examined the effect of different combinations of TGF-β1, miR-214-3p inhibitor and shLINC00665. The results revealed that the expression of XBP-1 was obviously downregulated after transfection with shLINC00665 after TGF-β1 compared to treatment with TGF-β1 alone; however, the effect of shLINC00665 was reversed by inhibiting miR-214-3p (Supplementary file Figure 3J, p<0.01). To summarize, these results demonstrated that LINC00665 could regulate the expression of XBP-1 by targeting miR-214-3p.

### Role of LINC00665 in BLM‑induced pulmonary fibrosis

HE staining was performed at 3 different times (7, 14, and 28 day) after BLM treatment to observe the pulmonary fibrosis in mouse lung tissue. Supplementary file Figure 4A provides the results of HE staining; the alveolar cavity structure of the control mice was intact, only a few inflammatory cells infiltrated in the interstitium, and the alveolar epithelium and the capillaries were normal. However, in the BLM-treated group, pulmonary fibrosis, structural disorder of bronchi and alveoli, chronic inflammatory interstitial infiltration, alveolar wall fracture and alveolar cavity fusion increased significantly. Moreover, we determined the LINC00665 mRNA expression in the NS or BLM group, and the expression of LINC00665 mRNA increased significantly in the BLM group compared to the control group at the same series of time points (Supplementary file Figure 4B, p<0.05 and p<0.01, respectively). The BLM-induced mouse pulmonary fibrosis was modelled by endotracheal administration of BLM under anesthesia, and normal saline solution was used as a control. According to the different treatment groups after modelling 72 h after injection, the transplantation group was intratracheally injected with shLINC00665, and the control group was injected with shNC. Next, we determined the expression of marker molecules (α-SMA, E-cadherin, collagen 1 and fibronectin) in each group. In the BLM-induced groups, the expression levels of α-SMA, collagen 1 and fibronectin were significantly increased in the merged processing groups with shNC compared with the groups combined with shLINC00665 (Supplementary file Figure 4C, p<0.05). The expression of E-cadherin was significantly decreased in the combined treatment of BLM with shNC group in contrast, its level was upregulated with shLINC00665 (Supplementary file Figure 4C, p<0.01). As shown in the HE and Masson staining images, treatment with shLINC00665 significantly reduced BLM-induced pulmonary fibrosis, and there was no difference in lung tissue between the saline-treated groups with or without shLINC00665 (Supplementary file Figures 4D and 4E). In summary, LINC00665 was highly expressed in BLM-induced lung tissues, and knockdown of LINC00665 alleviated BLM-induced pulmonary fibrosis.

## DISCUSSION

IPF occurs primarily in elderly subjects, and exposure to cigarette smoke is one of the main risk factors, which are associated with both pulmonary and extrapulmonary comorbidities^1,2^. We had retrospectively analyzed the case data of IPF patients diagnosed in our hospital, and found that the survival time of smoking IPF patients was shorter than that of non-smoking IPF patients, by univariate analysis. Although this was a single-center clinical study, it suggested that smoking is one of the important factors that may promote the process of IPF. CSE can promote AT2 cell senescence and pulmonary fibrosis^19^. Cigarette exposure in the environment may stimulate airway epithelial cells to promote pulmonary fibrosis^20^. However, the mechanism of the influence of smoking on the progression and prognosis of IPF is not very clear.

Next, we mined information related to smoking in the Biotrust database of IPF patients. First, we found that the expression of LINC00665 and XBP-1 in IPF lung tissues was obviously higher than that in normal healthy control lung tissues after data analysis. At the same time, we also found that LINC00665 and XBP-1 in the lung tissue of smokers were significantly higher than those in the lung tissue of non-smokers. The expression of LINC00665 in the lung tissue of smoker IPF patients is also significantly higher than that of healthy smoker controls. These results suggested that tobacco exposure may lead to increased expression of LINC00665 and XBP-1 in IPF patients, but the specific mechanism is still unclear.

Our study attempted to elucidate the possible mechanism by which CSE regulates LINC00665/XBP-1 in the process of pulmonary fibrosis through cell and animal experiments. CSE could promote the lung fibroblast-to-myofibroblast transition^15^, and at the same time, CSE can increase the expression level of LINC00665 in lung fibroblasts, and shLINC00665 can maintain the fibrogenic effect of CSE on lung fibroblasts and the expression of XBP-1. We revealed that LINC00665 may regulate the downstream target gene XBP-1 through miR-214-3p to affect the specific pathways of LFB proliferation. LINC00665 was also found to be highly expressed in BLM-induced pulmonary fibrosis in mice, and LINC00665 silencing had an inhibitory effect on BLM-induced mouse pulmonary fibrosis.

LncRNA expression is dysregulated in IPF, and dysregulated lncRNAs can be involved in the formation of IPF through the EMT pathway^3,4^. This is consistent with the fact that LINC00665 was significantly increased in the lung tissue of IPF patients compared with healthy controls in our database analysis. Our research demonstrated that CSE could increase the expression of LINC00665 in lung fibroblasts, and then increase the expression of XBP-1 and α-SMA. This fibrogenic effect of CSE could be inhibited by silencing LINC00665. These results suggest that LINC00665 can promote pulmonary fibrosis, which is also consistent with the results of the increase in LINC00665 in the smoker IPF lung tissues from the IPF database analysis. A few studies have found that miRNAs are crucial in the transformation of LFBs^6^, some of which are regulated by lncRNAs. In our study, we found that LINC00665 could bind to miR-214-3p and target and regulate it. In addition, XBP-1 has been identified as a target of miR-214-3p, so XBP-1 could be regulated by LINC00665 to act on the process of pulmonary fibrosis. This was consistent with the increasing trend of XBP-1 in IPF lung tissue and smoker lung tissue in the database.

UPR^ER^ matches the folding capacity of the ER to the load of client proteins in the organelle. XBP-1 is a direct target of IRE1 endonuclei activity in the UPR^ER^ activation pathway of the ER unfolded protein response triggered by ER stress and is a marker molecule for URP^ER^. ER stress participates in the occurrence and development of IPF and other fibrotic diseases^21^. Maladjustment of URP^ER^ was found in pulmonary fibroblasts and epithelial cells of patients with IPF^10^. Activation of URP^ER^ causes IRE1-dependent splicing of a small intron from XBP-1 mRNA^22^. In the pathogenesis of IPF, the pulmonary microenvironment of LFBs maintains a balanced state under normal physiological conditions, but under the action of external/endogenous pathogenic factors such as cigarette smoke, air pollution and aging, the microenvironment balance is destroyed, thus promoting the transformation of LFBs into MBs. XBP-1 is involved in the activation of fibroblasts^15,23,24^. Compared with that in non-IPF lungs, XBP-1 expression in IPF lungs was increased. Considering lung function independently, the increase in XBP-1 was related to the decrease in lung function (FEV_1_, FVC, TLC, RV)^25^. CSE can induce UPR^ER^ in lung fibroblasts, thereby increasing the expression level of XBP-1^15^. All of the above are consistent with our analysis results in the IPF database. However, the specific cellular and molecular mechanism of XBP-1 in pulmonary fibrosis is not very clear, and more clinical studies are needed to explore the relationship between XBP-1 and the decline in IPF pulmonary function.

In the next animal experiment, the expression of LINC00665 was markedly increased in the fibrotic lung tissues of the model group in a murine BLMinduced pulmonary fibrosis mouse model, and silencing LINC00665 antagonized the fibrogenic effect of BLM on the lung tissues of the mice. The inhibition of LINC00665 can inhibit the process of pulmonary fibrosis. In summary, LINC00665 silencing can improve pulmonary fibrosis in mice and inhibit the fibrogenic effect of CSE on pulmonary fibroblasts. However, more research is needed to demonstrate the specific mechanism of tobacco exposure and LINC00665 in vivo, and we need to continue to further study the feasibility of the animal model of lung fibrosis induced by cigarette smoke. More experiments are needed to further study this direction. Our results showed that CSE regulates LINC00665/XBP-1, which may play an important role in the process of pulmonary fibrosis and may become a new target for IPF treatment.

The differential expression of smoking-related lncRNAs and proteins in IPF patients was first identified using a public database, and then the mechanism of action of these in pulmonary fibrosis was verified in cell experiments and mouse models of pulmonary fibrosis. This is a limitation of this study, and we will continue to work to recommend better animal models for studying the relationship between smoking and pulmonary fibrosis.

## CONCLUSIONS

We found that smokers with IPF had a poor prognosis compared with non-smokers. After retrieval search analysis of the bioinformatics database, we also found that the expression of both LINC00665 and XBP-1 in IPF lung tissue and smoker lung tissue were significantly upregulated. Next, in the mice model of pulmonary fibrosis, we confirmed that CSE positively regulates LINC00665/XBP-1 to participate in the lung fibroblast-to-myofibroblast transition and that LINC00665 regulates XBP-1 by negatively regulating miR-214-3p. The inhibitory effect of silencing LINC00665 on pulmonary fibrosis in mice was also observed. Our study is the first to reveal that the pathogenesis of CSE regulates LINC00665/XBP-1 in the process of pulmonary fibrosis. Since it is not possible to fully simulate the mechanism of smoking on pulmonary fibrosis *in vivo*, more exploration work should be successfully performed to find better animal models of pulmonary fibrosis. More clinical trials are needed to validate the potential clinical utility of LINC00665/XBP-1 in the assessment of prognosis in smoker IPF patients. These results contribute to the basic theory of the pathogenesis of IPF and identify new target drugs for the prevention of pulmonary fibrosis.

## Data Availability

The data supporting this research are available from the authors on reasonable request.
