# Supplementary material for: CSE regulates LINC000665/XBP-1 in the progress of pulmonary fibrosis
Source: Tob Induc Dis. 2023 Dec 18;21:170. doi: 10.18332/tid/175004 (PMC10726211; doi:10.18332/tid/175004)
Supplement: Supplementary file 1 [file TID-21-170-s1.pdf]

Figure 1.The effect of smoking on the prognosis of IPF patients and the differentially expressed lncRNAs and proteins associated with smoking in IPF lung tissue: A)*The database included 46 IPF and 26 normal lung tissues;* ***B)*** *LINC00665 in the lung tissue of these IPF patients was obviously higher than that in normal healthy controls;* ***C)****XBP-1 in the lung tissue of these IPF patients was significantly higher than that in the control group;* ***D)****LINC00665 in the lung tissue of former smokers was obviously higher than that in never smokers;* ***E)*** *XBP-1 in the lung tissue of former smokers was significantly higher than that in never smokers;* ***F)*** *LINC00665 in the lung tissue of former smokers IPF patients was obviously higher than that in former smokers healthy controls.The statistics of the two-group comparisons were compared with the Wilcoxon test.*

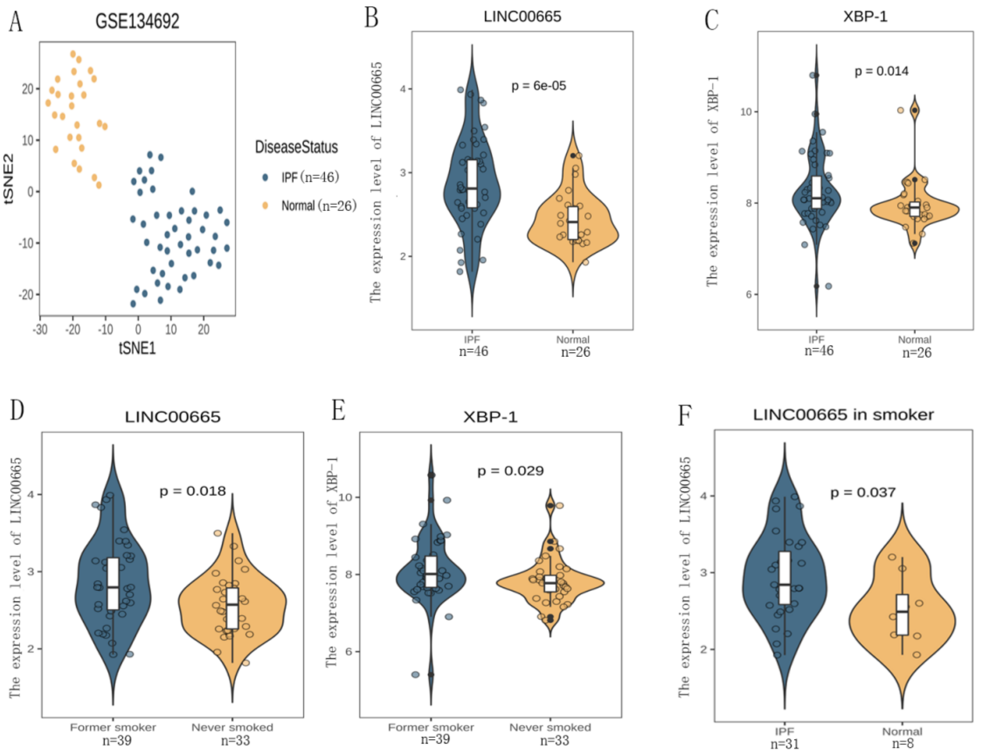

Figure 2. CSE regulates LINC00665 /XBP-1 in lung fibroblast-to-myofibroblast transition: A) *Immunocytochemical analysis of α-SMA protein expression at 0.1 M CSE and 1.0 M CSE;* ***B)*** *The expression of LINC00665 mRNA in the control group, 0.1 M CSE and 1.0 M CSE group at 24 h and 48 h time points;* ***C)*** *The expression of LINC00665 mRNA in fibroblasts after transfection with shRNA1 and shRNA2;* ***D)*** *Western blot and quantitative analysis of α-SMA and XBP-1 protein expression in different groups, control, CSE alone, CSE combined with shLINC00665 or shNC;* ***E)*** *The expression levels of α-SMA in these four groups were assessed by immunofluorescence;* ***F)*** *The protein expression levels of α-SMA and XBP-1 in these four groups were measured.*

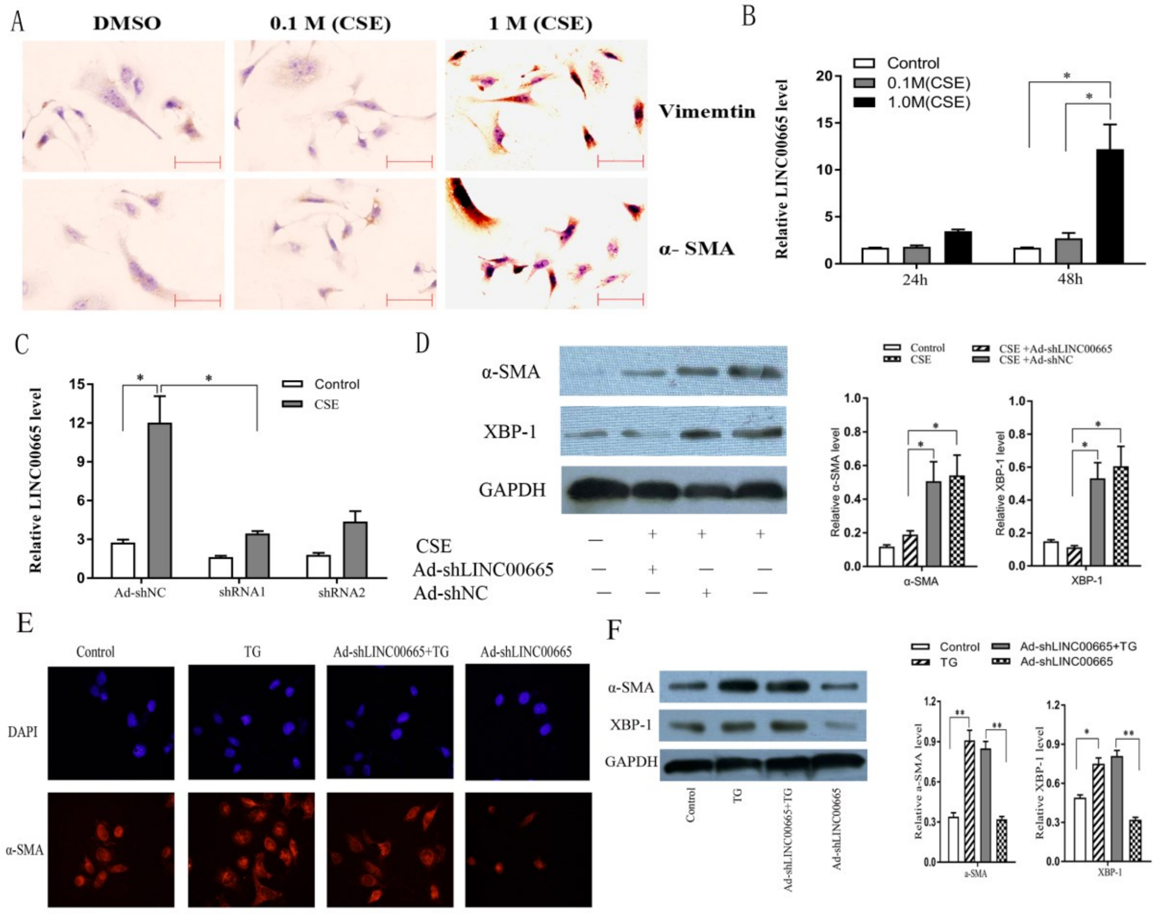

CSE: cigarette smoke extract; all the cells were obtained from the mouse model of pulmonary fibrosis.

Figure 3.LINC00665 regulated the expression of miR-214-3p, and could regulate the expression of XBP-1 by targeting miR-214-3p:A) *The binding sites in miR-214-3p between LINC00665 mRNA with wild-type (LINC00665 WT) and mutated-type (LINC00665 MUT) LINC00665 are shown;* ***B)*** *Dual-luciferase reporter assays were used to determine the luciferase activity of 293T cells cotransfected with miR-214-3p mimics or NC mimics and luciferase reporters containing LINC00665 WT or LINC00665 MUT;* ***C)*** *Determination of endogenous miR-214-3p precipitated by AGO2 upon overexpression of LINC00665 by RIP assay;* ***D)*** *Relative miR-214-3p levels in different groups were clarified;* ***E)*** *XBP-1 mRNA with wild-type (XBP-1 WT) and the mutated-type (XBP-1 MUT) in the miR-214-3p binding sites are displayed;* ***F)*** *LFBs were cotransfected with miR-214-3p mimics or NC mimics,and luciferase reporters containing the XBP-1 3'-UTR WT or 3'-UTR MUT transcript were resolved by dual-luciferase reporter assays;* ***G)*** *The mRNA expression levels of miR-214-3p in the miR-214-3p mimic,mimic-NC,inhibitor and inhibitor-NC groups are presented;* ***H)*** *The expression level of XBP-1 mRNA in the miR-214-3p mimic, inhibitor and their control groups was detected;* ***I)*** *Examination of the protein expression of XBP-1 in the miR-214-3p mimic,mimic-NC,inhibitor and inhibitor-NC groups;* ***J)*** *The protein expression level of XBP-1 was analysed by western blotting in the groups cotransfected with TGF-β1 and different combinations and cotransfected with shLINC00665 or its control (shNC) with or without miR-214-3p inhibitor.*

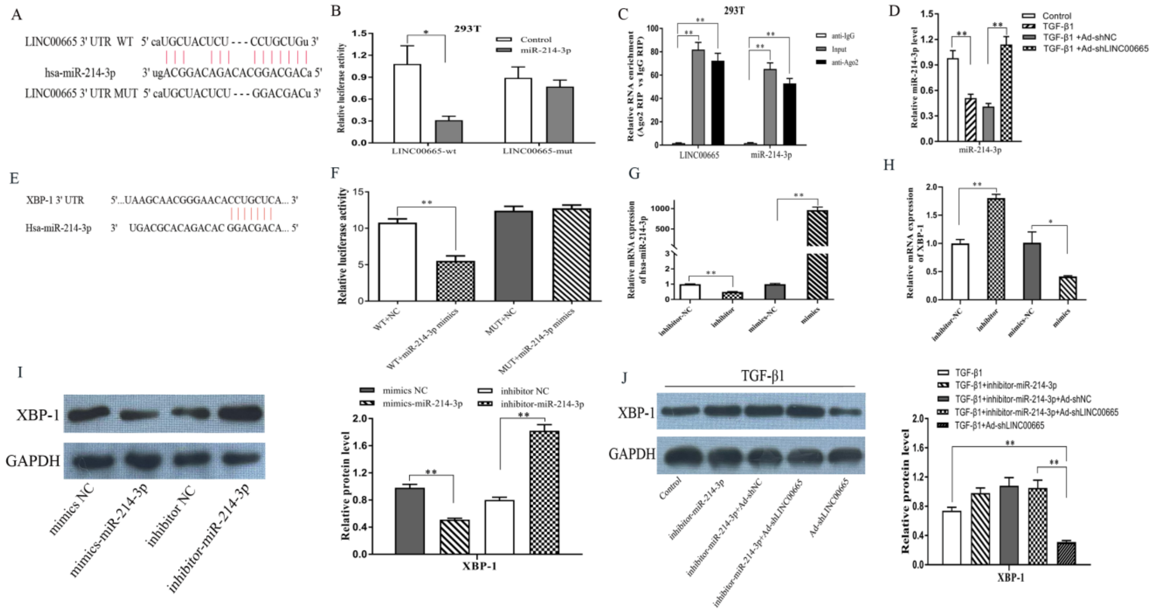

LFBs: lung fibroblasts;

Figure 4. LINC00665 was more highly expressed in BLM-induced mice pulmonary fibrotic lung tissues than in the NS-treated group, and knockdown of LINC00665 alleviated BLM-induced mice pulmonary fibrosis: A*) The pulmonary fibrosis of the BLM-induced group was evaluated by HE staining at 3 different time points (7, 14 and 28 days) after BLM injection; B) The expression level of LINC00665 was detected at 3 different time points (7,14 and 28 days) after BLM injection;* ***C****) The protein expression levels of α-SMA, collagen 1, fibronectin and E-cadherin in the NS- and BLM-treated groups with or without LINC00665 knockdown were tested by western blotting;* ***D)****The lung tissues in BLM or NS combined with different groups of shLINC00665 treatment or with shNC treatment were subjected to HE staining;* ***E****) The lung tissues in BLM or NS combined with different groups of shLINC00665 treatment or with shNC treatment were subjected to Masson staining.*

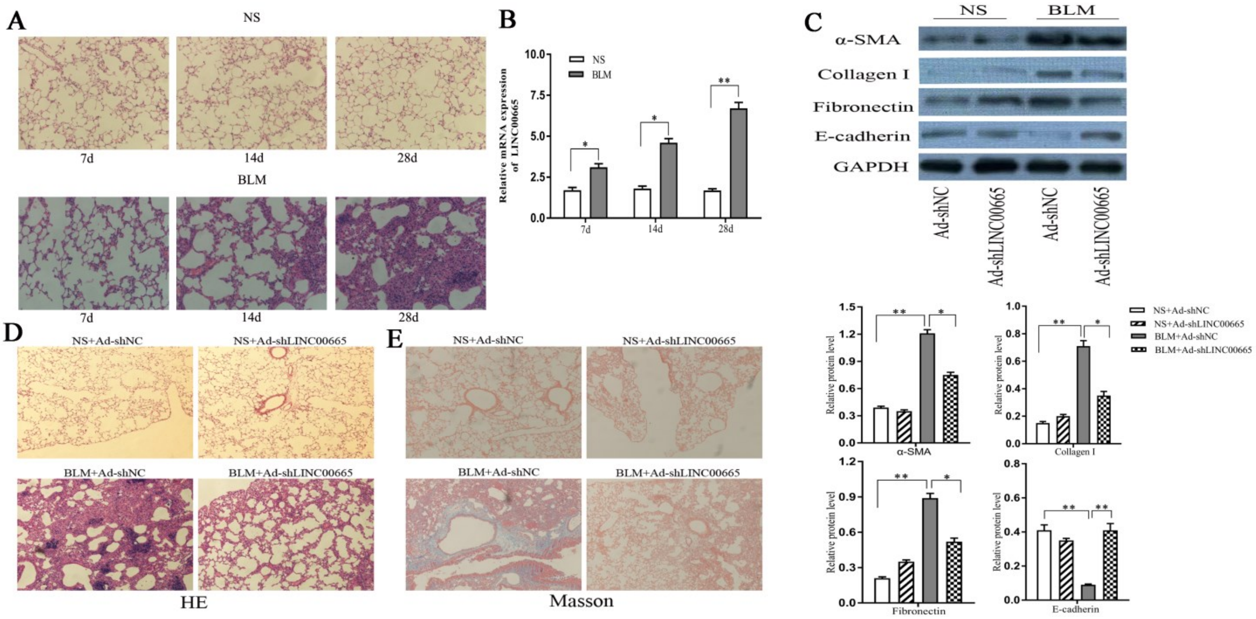

BLM:Bleomycin; NS:normal saline.

	sample_id	Batch	DiseaseSta	EP.Well	Sample.pr	SampleNa	Species	Tissue	Age	race	gender	tobacco	height	weight	sex
2	T_201708	2	Normal	EPE02P1	D3100002	normal_R	Human	Lung	75	Caucasian	Male	Never sm	72	231	M
3	T_201708	2	IPF	EPF01P1	R7100001	IPF_R6_B2	Human	Lung	70	Caucasian	Male	Former sm	69.01575	208.9982	M
4	T_201708	2	IPF	EPE01P1	R7100000	IPF_R4_B2	Human	Lung	60	Caucasian	Male	Never sm	64.48819	143.9619	M
5	m_201611	1	IPF	EPF06P1	R7100000	IPF_R24_	Human	Lung	60	African An	Male	Never sm	69.01575	156.0873	M
6	T_201708	2	Normal	EPC01P1	D3100003	normal_R	Human	Lung	20	Caucasian	Female	Never sm	62	104	F
8	T_201708	2	Normal	EPH01P1	D3100001	normal_R	Human	Lung	1	Caucasian	Female	Never sm	34.6	25	F
9	m_201611	1	IPF	EPE06P1	100750	IPF_R20_	Human	Lung	65	Caucasian	Male	Former sm	64.01575	123.2516	M
10	T_201708	2	Normal	EPD02P1	D3100002	normal_R	Human	Lung	15	African An	Female	Never sm	65	143	F
11	T_201708	2	Normal	EPD03P1	D3100001	normal_R	Human	Lung	20	Caucasian	Male	Never sm	71	165	M
12	T_201708	2	Normal	EPC03P1	D3100002	normal_R	Human	Lung	33	Caucasian	Male	Active sm	72	196	M
13	T_201708	2	Normal	EPC02P1	D3100003	normal_R	Human	Lung	30	Caucasian	Female	Former sm	68	121	F
15	T_201708	2	IPF	EPF02P1	R7100001	IPF_R7_B2	Human	Lung	50	Caucasian	Male	Former sm	70	186.0702	M
16	m_201611	1	Normal	EPB04P1	D3100002	Normal_R	Human	Lung	15	African An	Female	Never sm	65	143	F
17	m_201611	1	Normal	EPH07P1	D3100001	Normal_R	Human	Lung	1	Caucasian	Female	Never sm	34.6	25	F
18	T_201708	2	Normal	EPB02P1	D3100003	normal_R	Human	Lung	55	Caucasian	Male	Never sm	63	161	M
20	T_201708	2	IPF	EPA01P1	R7100000	IPF_R1_B2	Human	Lung	63	Caucasian	Male	Former sm	70	200.0012	M
21	m_201611	1	IPF	EPF07P1	R7100001	IPF_R25_	Human	Lung	69	Caucasian	Female	Former sm	64.01575	164.9939	F
24	m_201611	1	IPF	EPA07P1	R7100000	IPF_R2_B1	Human	Lung	65	Caucasian	Male	Former sm	69.29134	209.4391	M
25	m_201611	1	Normal	EPD04P1	D3100001	Normal_R	Human	Lung	55	African An	Male	Active sm	70	196	M
26	m_201611	1	IPF	EPA04P1	R7100001	IPF_R1_B1	Human	Lung	50	Caucasian	Male	Former sm	70	186.0702	M
27	m_201611	1	Normal	EPG07P1	D3100003	Normal_R	Human	Lung	20	Caucasian	Female	Never sm	62	104	F
28	m_201611	1	Normal	EPF05P1	D3100003	Normal_R	Human	Lung	1	Caucasian	Female	Never sm	33	29	F
29	m_201611	1	IPF	EPG04P1	R7100000	IPF_R27_	Human	Lung	53	Caucasian	Male	Never sm	28.26772	335.1026	M
31	m_201611	1	IPF	EPE01P1	RA100019	IPF_R17_	Human	Lung	71	Caucasian	Male	Former sm	72.00787	220.0213	M
32	T_201708	2	IPF	EPE04P1	R7100001	IPF_R5_B2	Human	Lung	69	Other	Male	Former sm	70	128.0004	M
33	T_201708	2	IPF	EPG02P1	R7100001	IPF_R9_B2	Human	Lung	64	Caucasian	Male	Former sm	70	197.9751	M
35	m_201611	1	IPF	EPD07P1	R7100001	IPF_R15_	Human	Lung	64	Caucasian	Male	Former sm	70	197.9751	M
36	m_201611	1	IPF	EPC08P1	RA100016	IPF_R13_	Human	Lung	59	Caucasian	Female	Former sm	62.99213	125.6635	F
37	m_201611	1	Normal	EPD06P1	D3100002	Normal_R	Human	Lung	15	African An	Female	Never sm	65	143	F
38	m_201611	1	IPF	EPG08P1	R7100001	IPF_R30_	Human	Lung	65	Caucasian	Female	Former sm	64.01575	162.0398	F
39	m_201611	1	IPF	EPA08P1	R7100000	IPF_R3_B1	Human	Lung	60	African An	Male	Never sm	70	128.0004	M
40	m_201611	1	Normal	EPC04P1	D3100003	Normal_R	Human	Lung	30	Caucasian	Female	Former sm	68	121	F
41	m_201611	1	IPF	EPD08P1	R7100000	IPF_R16_	Human	Lung	63	Caucasian	Male	Former sm	70	200.0012	M
42	m_201611	1	IPF	EPC03P1	R7100001	IPF_R10_	Human	Lung	65	Caucasian	Male	Never sm	72.04724	187.3929	M
43	m_201611	1	IPF	EPG06P1	100743	IPF_R29_	Human	Lung	60	Caucasian	Male	Never sm	67.99213	160.0556	M
44	m_201611	1	IPF	EPC02P1	R7100000	IPF_R9_B1	Human	Lung	66	Caucasian	Male	Never sm	72.00787	204.0025	M
45	m_201611	1	Normal	EPF08P1	D3100002	Normal_R	Human	Lung	33	Caucasian	Male	Active sm	72	196	M
46	m_201611	1	IPF	EPH05P1	R7100001	IPF_R35_	Human	Lung	52	Caucasian	Female	Former sm	69.01575	205.4708	F
47	m_201611	1	Normal	EPB06P1	D3100002	Normal_R	Human	Lung	75	Caucasian	Male	Never sm	72	231	M
48	m_201611	1	IPF	EPB03P1	R7100000	IPF_R5_B1	Human	Lung	60	Caucasian	Male	Never sm	67.51968	223.9897	M
49	m_201611	1	IPF	EPC07P1	R7100001	IPF_R12_	Human	Lung	64	Caucasian	Female	Former sm	65	126.9863	F
50	m_201611	1	Normal	EPF04P1	D3100002	Normal_R	Human	Lung	50	Caucasian	Male	Active sm	72	173	M
51	m_201611	1	IPF	EPE08P1	R7100001	IPF_R21_	Human	Lung	69	Other	Male	Former sm	70	128.0004	M
52	m_201611	1	IPF	EPF02P1	R7100000	IPF_R23_	Human	Lung	65	Caucasian	Male	Former sm	69.01575	205.0079	M
53	m_201611	1	IPF	EPB07P1	100749	IPF_R7_B1	Human	Lung	67	Caucasian	Male	Former sm	67.99213	190.5014	M
54	T_201708	2	IPF	EPF03P1	R7100001	IPF_R8_B2	Human	Lung	65	Caucasian	Female	Former sm	64.01575	162.0398	F
55	m_201611	1	IPF	EPB01P1	R7100000	IPF_R4_B1	Human	Lung	65	Caucasian	Male	Never sm	67.00787	173.9447	M
56	T_201708	2	IPF	EPA04P1	R7100001	IPF_R3_B2	Human	Lung	64	Caucasian	Female	Former sm	65	126.9863	F
57	m_201611	1	IPF	EPG03P1	R7100001	IPF_R26_	Human	Lung	58	Caucasian	Male	Never sm	72.00787	229.9421	M
58	T_201708	2	IPF	EPA03P1	R7100001	IPF_R2_B2	Human	Lung	69	Caucasian	Female	Former sm	64.01575	164.9939	F
59	m_201611	1	IPF	EPH04P1	RA100003	IPF_R34_	Human	Lung	48	African An	Female	Never sm	62.00787	180.0008	F
60	m_201611	1	Normal	EPA02P1	D3100001	Normal_R	Human	Lung	80	Caucasian	Female	Former sm	66	167	F
61	m_201611	1	IPF	EPC01P1	R7100001	IPF_R8_B1	Human	Lung	65	Other	Male	Former sm	65.98425	151.8985	M
62	m_201611	1	Normal	EPA01P1	D3100003	Normal_R	Human	Lung	1	Caucasian	Male	Never sm	35	37	M
63	m_201611	1	Normal	EPF03P1	D3100002	Normal_R	Human	Lung	38	Caucasian	Female	Active sm	68	220	F
64	m_201611	1	IPF	EPD03P1	R7100000	IPF_R14_	Human	Lung	64	Caucasian	Male	Never sm	26.37795	271.1686	M
65	T_201708	2	IPF	EPH02P1	R7100001	IPF_R10_	Human	Lung	65	Other	Male	Former sm	65.98425	151.8985	F
66	m_201611	1	IPF	EPH01P1	100742	IPF_R31_	Human	Lung	64	Caucasian	Female	Former sm	65.35433	180.7791	F
67	m_201611	1	Normal	EPD01P1	D3100001	Normal_R	Human	Lung	20	Caucasian	Male	Never sm	71	165	M
68	m_201611	1	IPF	EPE05P1	R7100001	IPF_R19_	Human	Lung	56	Caucasian	Female	Former sm	62.99213	115.0813	F
69	m_201611	1	IPF	EPB05P1	RA100008	IPF_R6_B1	Human	Lung	66	Caucasian	Male	Former sm	67.99213	166.0081	M
70	m_201611	1	IPF	EPC05P1	R7100000	IPF_R11_	Human	Lung	64	Caucasian	Male	Former sm	69.01575	139.1117	M
71	m_201611	1	IPF	EPG05P1	R7100001	IPF_R28_	Human	Lung	71	Other	Male	Never sm	70	121.9156	M
72	m_201611	1	IPF	EPH02P1	RA100017	IPF_R32_	Human	Lung	66	Caucasian	Male	Former sm	67.51968	179.8972	M
73	m_201611	1	Normal	EPG02P1	D3100002	Normal_R	Human	Lung	53	Caucasian	Female	Never sm	62	174	U
74	m_201611	1	Normal	EPA05P1	D3100002	Normal_R	Human	Lung	2	Unknown	Male	Never sm	29	20	M
75	m_201611	1	Normal	EPH08P1	D3100003	Normal_R	Human	Lung	55	Caucasian	Male	Never sm	63	161	M
76	m_201611	1	IPF	EPH03P1	R7100000	IPF_R33_	Human	Lung	67	Caucasian	Male	Never sm	69.01575	185.0119	M
77	m_201611	1	IPF	EPE02P1	R7100001	IPF_R18_	Human	Lung	64	Caucasian	Male	Former sm	72.00787	223.9897	M
78	m_201611	1	IPF	EPF01P1	R7100001	IPF_R22_	Human	Lung	70	Caucasian	Male	Former sm	69.01575	208.9982	M
79	m_201611	1	IPF	EPH06P1	R7100000	IPF_R36_	Human	Lung	60	Caucasian	Male	Never sm	64.48819	143.9619	M
80	m_201611	1	Normal	EPE03P1	D3100001	Normal_R	Human	Lung	54	Caucasian	Male	Never sm	70	189	M

© 2023 Song M. et al.
